# Incorporating polygenic risk into the Leicester Risk Assessment score for 10-year risk prediction of type 2 diabetes

**DOI:** 10.1016/j.dsx.2024.102996

**Published:** 2024-04

**Authors:** Xiaonan Liu, Thomas J. Littlejohns, Jelena Bešević, Fiona Bragg, Lei Clifton, Jennifer A. Collister, Eirini Trichia, Laura J. Gray, Kamlesh Khunti, David J. Hunter

**Affiliations:** a Nuffield Department of Population Health, University of Oxford, Oxford, UK; b MRC Population Health Research Unit, Nuffield Department of Population Health, University of Oxford, Oxford, UK; c Department of Population Health Sciences, University of Leicester, UK; d Diabetes Research Centre, University of Leicester, Leicester, UK; e Department of Epidemiology, Harvard T.H. Chan School of Public Health, Boston, USA

**Keywords:** Type 2 diabetes, Polygenic risk score, Risk prediction, Leicester Risk Assessment, UK Biobank

## Abstract

**Aims:**

We evaluated whether incorporating information on ethnic background and polygenic risk enhanced the Leicester Risk Assessment (LRA*)* score for predicting 10-year risk of type 2 diabetes.

**Methods:**

The sample included 202,529 UK Biobank participants aged 40–69 years. We computed the *LRA score*, and developed two new risk scores using training data (80% sample): *LRArev*, which incorporated additional information on ethnic background, and *LRAprs*, which incorporated polygenic risk for type 2 diabetes. We assessed discriminative and reclassification performance in a test set (20% sample). Type 2 diabetes was ascertained using primary care, hospital inpatient and death registry records.

**Results:**

Over 10 years, 7,476 participants developed type 2 diabetes. The Harrell's C indexes were 0.796 (95% Confidence Interval [CI] 0.785, 0.806), 0.802 (95% CI 0.792, 0.813), and 0.829 (95% CI 0.820, 0.839) for the *LRA*, *LRArev* and *LRAprs* scores, respectively. The *LRAprs* score significantly improved the overall reclassification compared to the *LRA* (net reclassification index [NRI] = 0.033, 95% CI 0.015, 0.049) and *LRArev* (NRI = 0.040, 95% CI 0.024, 0.055) *scores*.

**Conclusions:**

Polygenic risk moderately improved the performance of the existing *LRA score* for 10-year risk prediction of type 2 diabetes.

## Highlights

• The original LRA score performed well at predicting risk of type 2 diabetes.
• Incorporation of polygenic risk resulted in a moderate improvement of type 2 diabetes risk prediction.
• The LRA score with polygenic risk performed better at correctly classifying future cases.
• Conversely, the LRA score with polygenic risk classified a higher proportion of non-cases during follow-up as high-risk.

## 1 Introduction

The prevalence of type 2 diabetes has rapidly increased in recent decades, with an estimated half a billion individuals worldwide currently living with the condition [1]. However, type 2 diabetes is largely preventable and risk prediction tools play a key role in the identification of high-risk individuals who can be targeted for risk reduction strategies. Existing tools include a combination of lifestyle, clinical and/or biochemical information [2]. In recent years, hundreds of genetic variants associated with type 2 diabetes risk have been identified, and these can be combined to produce an individual's polygenic risk of type 2 diabetes. Given the heritability of type 2 diabetes, it is important to understand whether incorporating genetic risk alongside non-genetic factors improves the predictive performance of type 2 diabetes risk tools [3]. Previous studies have derived new risk scores for type 2 diabetes [[4], [5], [6], [7], [8], [9], [10], [11]], however there is a lack of research investigating whether existing risk prediction tools are enhanced once genetic factors are incorporated into the models.

The Leicester Risk Assessment (LRA) is a simple, non-invasive score designed to identify individuals at high risk of non-diabetic hyperglycaemia (also known as prediabetes) or undiagnosed type 2 diabetes risk based on seven self-reported questions [12]. The *LRA score* was published in 2010 using data from a cross-sectional multi-ethnic UK population of 40–74 year olds. The *LRA score* has also been shown to accurately predict risk of self-reported type 2 diabetes within 10 years [13]. As a result, the *LRA score* has been recommended by the National Institute for Health and Care Excellence (NICE) [14,15] as a tool for individuals to assess their future risk of type 2 diabetes. The *LRA score* is also available on the website of a charitable organisation, Diabetes UK, and has been completed almost 3.0 million times by April 2024 [16].

In the current study, we investigated the predictive performance of the *LRA score* with incorporation of more detailed information on ethnic background and polygenic risk for type 2 diabetes for 10-year risk of developing incident type 2 diabetes in a UK-based cohort of 210,000 participants aged 40–69 years.

## 2 Subjects

The UK Biobank (UKB) population-based cohort recruited half a million women and men aged 40–69 years between 2006 and 2010 [17]. All participants attended one of 22 baseline assessment centres located throughout England, Scotland and Wales and provided informed consent. At baseline assessment, participants provided sociodemographic, lifestyle and health-related information via a touchscreen questionnaire and nurse-led verbal interview, underwent a range of physical examinations and provided biological samples, including blood. Incident disease diagnoses were captured through cohort-wide linkage to hospital inpatient and death registry records and with primary care records available for approximately 45% of participants through 2017. UKB received ethical approval from the National Health Service North West Centre for Research Ethics Committee (Ref: 11/NW/0382).

A validation study found that only 41% of individuals with a diagnosis of type 2 diabetes in primary care have a corresponding diagnosis in their secondary care record [18]. Recently, we also demonstrated that primary care captures the vast majority type 2 diabetes cases in UKB [19]. Therefore, in the current study, we restricted our population to the 234,209 UKB participants with available longitudinal primary care records. We further excluded participants with missing genetic and phenotypic data used to derive the risk scores for type 2 diabetes, or with prevalent non-gestational diabetes (see Supplementary Fig. 1 for flow diagram).

## 3 Materials and methods

### 3.1 LRA scores for type 2 diabetes

We computed the original *LRA score* and developed two new risk scores: 1) a ‘revised’ *LRA score* which incorporated additional information on ethnic background, hereafter referred to as ‘*LRArev score’*, 2) and a ‘*LRAprs score’* which further included polygenic risk for type 2 diabetes into the *LRArev score*.

The *LRA score* was calculated from seven variables and is based on the same scoring system as the original score [12]. Age in years (40–49, 50–59, 60–69) and sex (Male, Female) were acquired from central registry records by UKB and confirmed by participants at baseline assessment. Ethnicity (White, Other), first degree family history of type 2 diabetes (No, Yes), and antihypertensive medication use or high blood pressure (No, Yes) were self-reported during the touchscreen questionnaire. Body mass index (BMI) in kg/m^2^ (<25, 25–29, 30–34, ≥35) and waist circumference in cm (<90, 90–99, 100–109, ≥110) were measured during the physical examination. For the original *LRA score*, a logistic regression model was run, with all seven variables entered as predictors and non-diabetic hyperglycaemia/undiagnosed type 2 diabetes as the outcome [12]. Scores were assigned to each category within the predictors based on the coefficients obtained from the models. For instance, compared to ‘White’ ethnicity, the coefficient for ‘Other’ ethnicity was ‘0.57’, which was rounded to a score of ‘6’ [12]. Then, the scores for each variable were added together to produce a total score ranging from 0 to 47, with a higher score indicating a greater risk.

For the *LRArev score, s*ix of the seven variables remained the same as the *LRA score*, except ethnicity. This was expanded from White and Other to include the following categories: White, Black, South Asian, Mixed, Other. The *LRAprs score* included polygenic risk for type 2 diabetes in addition to the seven variables as categorised in the *LRArev score.* The polygenic risk score (PRS) for type 2 diabetes was developed on external multi-ethnic genome-wide association study data and provided to UKB by Genomics PLC. The PRS was calculated as the sum of the per-variant effect size multiplied by allele dosage, followed by centering and variance-standardisation by ancestry. Genomics PLC excluded participants who were sex discordant, as well as outliers for genotype missingness or heterozygosity. We categorised the PRS into quintiles, with a higher quintile indicating a greater risk of developing type 2 diabetes.

### 3.2 Diabetes

Prevalent non-gestational diabetes was identified through (1) self-reported diagnosis or medication usage based on a previously published algorithm to ascertain diabetes in UKB [18], (2) hospital inpatient records, and (3) primary care diagnosis and medication records with date of diagnoses or prescription preceding or on the date of baseline recruitment. Incident type 2 diabetes was ascertained using hospital inpatient, death registry and primary care diagnosis records. Primary and secondary hospital diagnoses and causes of deaths were recorded using the International Classification of Diseases-10 (ICD-10) coding systems. Clinical events were recorded using Read version 2 and version 3 codes in the primary care data. The clinical codes used to ascertain diabetes and type 2 diabetes were selected based on a previously published definition of type 2 diabetes in UKB [20] are listed in Supplementary Table 1.

### 3.3 Statistical analyses

The sample was randomly split into training (80%, N = 162,023) and test (20%, N = 40,506) datasets. Model performance was assessed using the training and test datasets separately, with the training dataset used for obtaining model coefficients and the test dataset for validating how well the model generalises to unseen data. We performed two Cox proportional-hazards regression analyses to obtain the beta coefficients for the variables included in the 1) *LRArev score* and 2) *LRAprs score* in association with incident type 2 diabetes. The follow-up time in years was calculated from date of attending baseline assessment until date of first type 2 diabetes event (diagnosis and death), date of death from other causes, date lost to follow-up, GP deduction date in most recent practice or end of follow-up, whichever occurred first. End of follow-up was based on medical record data availability for each data provider, which was May 25, 2017 for Vision in England, May 31, 2016 for TPP in England, March 31, 2017 for Vision/EMIS in Scotland, and August 31, 2017 for Vision/EMIS in Wales. The proportionality assumption was visually inspected using the scaled Schoenfeld residuals. As with the original *LRA score*, the beta coefficients were multiplied by 10, rounded up to the nearest integer, and then summed to give the total risk score [12].

We assessed the discrimination of the risk scores for predicting 10 year risk of developing incident type 2 diabetes using the Harrell's C index. Harrell's *C*-index provides an indicator of how well a model discriminates between those who do, and do not, develop the outcome, with a score of 0.5 indicating a performance no better than chance [21]. Sensitivity and specificity was obtained for incident type 2 diabetes status for each cut-off point on the risk scores. We then calculated the categorical net reclassification index (NRI) [22] (see Supplementary Material for detailed NRI methods) to assess the reclassification of 10 year risk for each of the three pairwise comparisons: 1) *LRArev score* vs *LRA score*, 2) *LRAprs score* vs *LRArev score*, and 3) *LRAprs score* vs *LRA score*. The corresponding 95% confidence interval (CI) was estimated using the bootstrap method with 1000 samples*.* NRI quantifies changes in risk prediction by assessing how a new model reclassifies cases and controls compared to an original model based on a given risk threshold [23]. Typically, computation of categorical NRI involves applying the same cut-off as the original score to the new risk scores. For the original *LRA,* a score of 16 is the established clinically actionable threshold [12]. Since the new risk scores have different scales, we derived the optimal cut-offs for the *LRArev score* and *LRAprs score* based on percentiles using the training data. We obtained the percentage of individuals with a *LRA score* below 16 and applied the same percentile cut-point to the *LRArev score* and *LRAprs score*.

Calibration of the three risk scores with 10-year risk of incident type 2 diabetes was assessed by comparing the observed and predicted risks across quintiles of predicted risk. For *LRA score*, we calculated the predicted risk as the probability of having impaired glucose regulation or type 2 diabetes at baseline using the intercept and model coefficients (Supplementary Table 2). For the two new risk scores, we computed the predicted risks as the probability of developing type 2 diabetes within 10 years using baseline hazard and model coefficients derived from the training data. Since the maximum follow-up of one quintile group was less than 10 years, we computed the observed 9-year type 2 diabetes risk using the Kaplan–Meier estimator as an approximation of the observed 10-year risk.

In a sensitivity analysis, we re-developed *LRArev* and *LRAprs* scores using only the primary care records to ascertain type 2 diabetes, and assessed the model performances using the methods described above. In addition, since those with previous gestational diabetes typically follow an alternative screening route, we further excluded prevalent gestational diabetes at baseline from the study population as a sensitivity analysis. Furthermore, we investigated the performance of the scores in females and males separately. All statistical tests were two-tailed, at a 5% significance level.

All analyses were performed using R version 4.2.2. The following R packages were used in the model validation: *timeROC* was used to produce time-dependent ROC curves, *Hmisc* was used to compute the Harrell's *C*-index, *caret* was used to compute sensitivity and specificity, and *nricens* was used to compute NRI.

## 4 Results

### 4.1 Baseline characteristics

The final sample included 202,529 participants. Of these, 7,476 (3.7%) participants developed incident type 2 diabetes over a 10-year follow-up period (median follow-up = 7.1 years). Baseline characteristics by incident type 2 diabetes status are provided in Table 1. Participants who developed type 2 diabetes were more likely to be older, male, of non-White ethnic background, have a family history of diabetes, have a higher waist circumference and BMI, use antihypertensive medications or have high blood pressure, and higher type 2 diabetes PRS. A total of 162,023 (N = 6,040 cases) and 40,506 (N = 1,436 cases) participants were included in the training and test data sets, respectively (Supplementary Table 3).

**Table 1:** Baseline characteristics by incident type 2 diabetes status^a^.

Characteristics	Type 2 diabetes, N (%)		Total (N = 202,529)
	No (N = 195,053)	Yes (N = 7,476)	
Age in years			
40-49	47,397 (24.3)	1,101 (14.7)	48,498 (23.9)
50-59	66,117 (33.9)	2,458 (32.9)	68,575 (33.9)
60-69	81,539 (41.8)	3,917 (52.4)	85,456 (42.2)
Female	109,435 (56.1)	3,113 (41.6)	112,548 (55.6)
Self-reported ethnicity			
White	187,334 (96.0)	6,729 (90.0)	194,063 (95.8)
Black	1,863 (1.0)	166 (2.2)	2,029 (1.0)
South Asian	2,579 (1.3)	346 (4.6)	2,925 (1.4)
Mixed	972 (0.5)	54 (0.7)	1,026 (0.5)
Other	2,305 (1.2)	181 (2.4)	2,486 (1.2)
Family history of diabetes	38,446 (19.7)	2,583 (34.6)	41,029 (20.3)
Waist circumference in cm			
<90	102,072 (52.3)	1,168 (15.6)	103,240 (51.0)
90-99	53,417 (27.4)	2,017 (27.0)	55,434 (27.4)
100-109	27,908 (14.3)	2,333 (31.2)	30,241 (14.9)
≥110	11,656 (6.0)	1,958 (26.2)	13,614 (6.7)
Body Mass Index in kg/m^2^			
<25	67,275 (34.5)	604 (8.1)	67,879 (33.5)
25-29	84,777 (43.5)	2,597 (34.7)	87,374 (43.1)
30-34	32,256 (16.5)	2,582 (34.5)	34,838 (17.2)
≥35	10,745 (5.5)	1,693 (22.6)	12,438 (6.1)
Self-reported antihypertensive medication use or high blood pressure	50,257 (25.8)	3,824 (51.2)	54,081 (26.7)
Type 2 diabetes PRS in quintiles			
1, lowest	40,065 (20.5)	496 (6.6)	40,561 (20.0)
2	39,569 (20.3)	892 (11.9)	40,461 (20.0)
3	39,170 (20.1)	1,292 (17.3)	40,462 (20.0)
4	38,728 (19.9)	1,800 (24.1)	40,528 (20.0)
5, highest	37,521 (19.2)	2,996 (40.1)	40,517 (20.0)

Abbreviations: N, Number, PRS, Polygenic Risk Score.

a Median follow-up of 7.1 years.

### 4.2 Score development using training data

Table 2 shows the model output and scores for the *LRArev* and *LRAprs scores* obtained using the training data alongside the original scores for the *LRA score*. For both the *LRArev* and *LRAprs scores*, all categories within each variable were significantly associated with type 2 diabetes risk (p < 0.001). For the *LRArev score*, the categories within the expanded definition of ethnicity were associated with a higher risk of type 2 diabetes compared to White ethnicity: Black (Hazard Ratio [HR] = 2.33, 95% confidence interval [CI] 1.95, 2.77), South Asian (HR = 3.83, 95% CI 3.39, 4.32), Mixed (HR = 2.09, 95% CI 1.56, 2.80), and Other (HR = 2.76, 95% CI 2.34, 3.26). For the *LRAprs score*, a dose-response association was observed between PRS for type 2 diabetes quintiles and risk of type 2 diabetes.

**Table 2:** Cox proportional-hazards models for Type 2 diabetes in the training dataset and scoring system for risk scores.

Characteristic	LRA score	LRArev score			LRAprs score		
	Score^a^	HR (95% CI)	β (95% CI)^b^	Score	HR (95% CI)	β (95% CI)^b^	Score
Age in years							
40-49	0	Reference	Reference	0	Reference	Reference	0
50-59	5	1.47 (1.35, 1.59)	0.38 (0.30, 0.46)	4	1.52 (1.41, 1.65)	0.42 (0.34, 0.50)	4
60-69	9	1.88 (1.74, 2.03)	0.63 (0.55, 0.71)	6	2.02 (1.87, 2.18)	0.70 (0.63, 0.78)	7
Sex							
Female	0	Reference	Reference	0	Reference	Reference	0
Male	1	1.17 (1.10, 1.24)	0.15 (0.09, 0.21)	2	1.19 (1.12, 1.27)	0.18 (0.11, 0.24)	2
Self-reported ethnicity							
White	0	Reference	Reference	0	Reference	Reference	0
Non-White	6	–	–	–	–	–	–
Black	–	2.33 (1.95, 2.77)	0.84 (0.67, 1.02)	8	2.35 (1.97, 2.80)	0.85 (0.68, 1.03)	9
South Asian	–	3.83 (3.39, 4.32)	1.34 (1.22, 1.46)	13	4.04 (3.58, 4.57)	1.40 (1.28, 1.52)	14
Mixed	–	2.09 (1.56, 2.80)	0.74 (0.45, 1.03)	7	2.02 (1.51, 2.70)	0.70 (0.41, 0.99)	7
Other	–	2.76 (2.34, 3.26)	1.02 (0.85, 1.18)	10	2.83 (2.39, 3.34)	1.04 (0.87, 1.21)	10
Family history of diabetes							
No	0	Reference	Reference	0	Reference	Reference	0
Yes	5	1.89 (1.79, 2.00)	0.64 (0.58, 0.69)	6	1.68 (1.59, 1.77)	0.52 (0.46, 0.57)	5
Waist circumference in cm							
<90	0	Reference	Reference	0	Reference	Reference	0
90-99	4	1.94 (1.76, 2.13)	0.66 (0.57, 0.76)	7	1.89 (1.72, 2.08)	0.64 (0.54, 0.73)	6
100-109	6	3.16 (2.84, 3.53)	1.15 (1.04, 1.26)	12	3.07 (2.75, 3.42)	1.12 (1.01, 1.23)	11
≥110	9	4.51 (3.96, 5.13)	1.51 (1.38, 1.64)	15	4.39 (3.86, 5.00)	1.48 (1.35, 1.61)	15
Body Mass Index in kg/m^2^							
<25	0	Reference	Reference	0	Reference	Reference	0
25-29	3	1.81 (1.62, 2.02)	0.59 (0.48, 0.70)	6	1.73 (1.55, 1.93)	0.55 (0.44, 0.66)	5
30-34	5	2.77 (2.45, 3.15)	1.02 (0.89, 1.15)	10	2.58 (2.28, 2.93)	0.95 (0.82, 1.07)	9
≥35	8	3.81 (3.29, 4.41)	1.34 (1.19, 1.48)	13	3.54 (3.06, 4.10)	1.26 (1.12, 1.41)	13
Self-reported antihypertensive medication use or high blood pressure							
No	0	Reference	Reference	0	Reference	Reference	0
Yes	5	1.70 (1.61, 1.79)	0.53 (0.48, 0.58)	5	1.64 (1.56, 1.73)	0.50 (0.44, 0.55)	5
Type 2 diabetes PRS in quintiles							
1, lowest	–			–	Reference	Reference	0
2	–			–	1.62 (1.44, 1.83)	0.48 (0.36, 0.61)	5
3	–			–	2.22 (1.98, 2.49)	0.80 (0.68, 0.91)	8
4	–			–	2.93 (2.63, 3.28)	1.08 (0.97, 1.19)	11
5, highest	–			–	4.63 (4.17, 5.15)	1.53 (1.43, 1.64)	15

Abbreviations: CI, Confidence Interval, PRS, Polygenic Risk Score, HR, Hazard Ratio.

The original Leicester score includes a score of 13 assigned to 70–75 year olds. As the upper age limit in UKB is 69 years, this age group was not included in the model.

a The scoring system of used for the *LRA score* was originally published elsewhere [12].

b The β is the log (Hazard ratio) and all categories within each variable were statistically significantly associated with type 2 diabetes risk (p < 0.001).

The sensitivity and specificity across all cut-points for *LRArev* and *LRAprs* computed using the training data are provided in Supplementary Table 4. The optimal threshold for predicting 10-year type 2 diabetes risk was identified as 20 (<20: low risk, ≥20: high risk) for the *LRArev score*, with sensitivity = 0.84 and specificity = 0.61. The optimal threshold for the *LRAprs score* was 27 (<27: low risk, ≥27: high risk) with sensitivity = 0.88 and specificity = 0.60. Both cut-points had balanced sensitivity and specificity, with higher sensitivity compared to that of *LRA score* using the established threshold of 16 (<16: low risk, ≥16: high risk), sensitivity = 0.82 and specificity = 0.62.

### 4.3 Discrimination, reclassification, and calibration

**Fig. 1:**
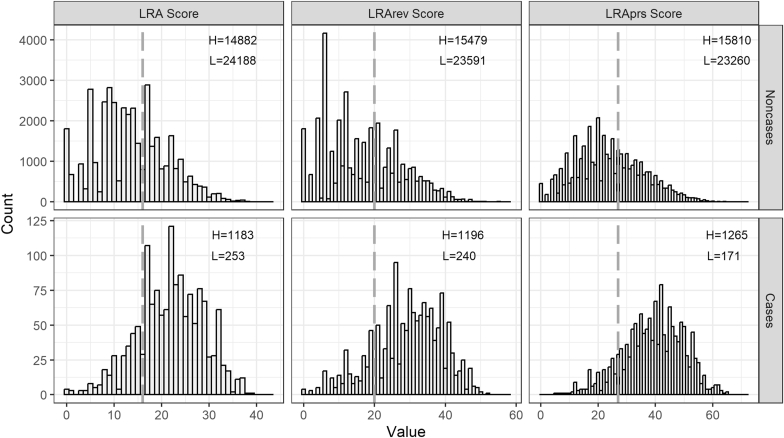
Distribution of three risk scores by Type 2 diabetes status in the test data. Abbreviations: L, number of people in low risk group, H, number of people in high risk. The dashed line represents the optimal cut-off value for each score: 16 for the *LRA score*, 20 for the *LRArev score*, and 27 for the enhanced score with *LRAprs score.* Individuals with risk scores below the cut-off were categorised as “Low risk” and vice versa. Mean scores were 13.8 (SD = 7.6), 17.6 (SD = 10.9) and 24.6 (SD = 12.1) for the *LRA*, *LRArev* and *LRAprs scores*, respectively.

Fig. 1 shows the distribution of three scores by type 2 diabetes in the test data. The total scores ranged from 0 to 43 for the *LRA score*, 0 to 58 for the *LRArev score* and from 0 to 72 for the *LRAprs score*. Mean scores were 13.8 (Standard Deviation [SD] = 7.6), 17.6 (SD = 10.9) and 24.6 (SD = 12.1) for the *LRA*, *LRArev* and *LRAprs scores*, respectively in the test data. Similar distributions and summary statistics of the three scores were observed in the training data (Supplementary Fig. 2).

**Fig. 2:**
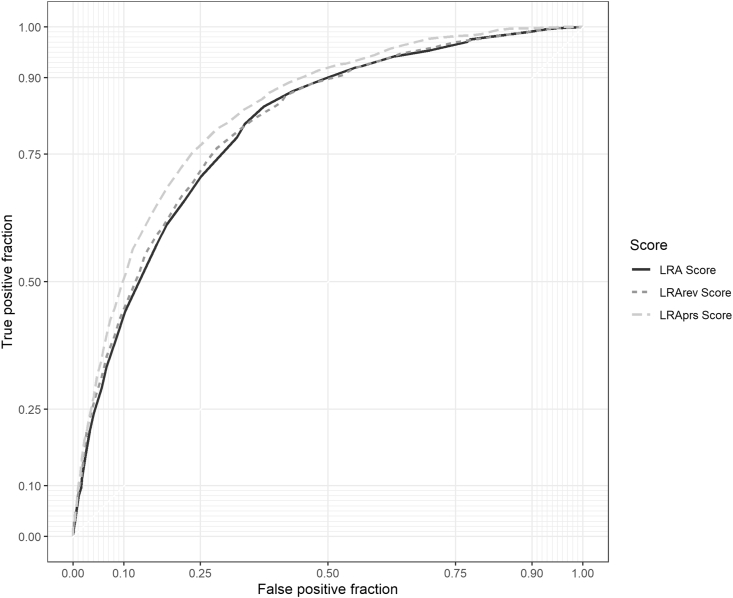
Time dependent ROC Curves for Type 2 diabetes using the risk scores in the test data^a^ ^a^ ROC curves plotted at 9 years due to low number of incident cases between 9 and 10 years follow-up.

In the test data, the *LRA score* showed good discrimination performance in predicting 10 year risk of type 2 diabetes (Harrell's C = 0.796, 95% CI 0.785, 0.806). In comparison to the *LRA score*, the *LRArev score* showed similar discrimination (Harrell's C = 0.802, 95% CI 0.792, 0.813) and the *LRAprs score* exhibited better discrimination (Harrell's C = 0.829, 95% CI 0.820, 0.839) (Table 3, Fig. 2).

**Table 3:** Harrell's C statistic for type 2 diabetes using the risk scores in the test data.

	Harrell's C statistic (95% CI)
LRA score	0.796 (0.785, 0.806)
LRArev score	0.802 (0.792, 0.813)
LRAprs score	0.829 (0.820, 0.839)

Abbreviations: CI, Confidence Interval, PRS, Polygenic Risk Score.

We computed the reclassification statistics and categorical NRI of 10-year risk for three sets of pairwise comparisons: 1) *LRArev score* vs *LRA score*, 2) *LRAprs score* vs *LRArev score*, and 3) the *LRAprs score* vs *LRA score* (Table 4). The reclassification of comparison 1 was not significantly different in the test data (overall NRI = −0.006, 95% CI -0.022, 0.009). In contrast, we observed positive and statistically significant improvements in reclassification of comparison 2 (overall NRI = 0.040, 95% CI 0.024, 0.055) and comparison 3 (overall NRI = 0.033, 95% CI 0.015, 0.049). The results for discrimination and reclassification were very similar in the training data (Supplementary Tables 5 and 6).

**Table 4:** Reclassification tables of A) LRArev score vs LRA score, B) LRAprs score vs LRArev score, and C) the LRAprs score vs LRA score, for risk of type 2 diabetes in the test data.

A)	LRA score	LRArev score		NRI (95% CI)	Overall NRI (95% CI)
		*Low risk*	*High risk*		
Cases	*Low risk*	186	67	0.009 (−0.007, 0.024)	−0.006 (−0.022, 0.009)
	*High risk*	54	1,129		
Noncases	*Low risk*	21,858	2,330	−0.015 (−0.018, −0.012)	
	*High risk*	1,733	13,149		

B)	LRArev score	LRAprs score		NRI (95% CI)	Overall NRI (95% CI)
		*Low risk*	*High risk*		
Cases	*Low risk*	145	95	0.048 (0.034, 0.063)	0.040 (0.024, 0.055)
	*High risk*	26	1,170		
Noncases	*Low risk*	20,959	2,632	−0.008 (−0.012, −0.005)	
	*High risk*	2,301	13,178		

C)	LRA score	LRAprs score		NRI (95% CI)	Overall NRI (95% CI)
		*Low risk*	*High risk*		
Cases	*Low risk*	136	117	0.057 (0.040, 0.073)	0.033 (0.015, 0.049)
	*High risk*	35	1,148		
Noncases	*Low risk*	20,703	3,485	−0.024 (−0.028, −0.020)	
	*High risk*	2,557	12,325		

Abbreviations: CI, Confidence Interval, NRI, Net Reclassification Index, PRS, Polygenic Risk Score.

We then assessed the calibration by plotting the observed risk across quintiles of predicted risk. The *LRA score* showed overestimation of 10-year risk of type 2 diabetes whereas the *LRArev score* and *LRAprs score* showed good calibration in the training and test data. (Supplementary Fig. 3).

We observed approximately 91.4% of the incident type 2 diabetes cases were captured in primary care in the training data (Supplementary Fig. 4). Sensitivity analyses deriving type 2 diabetes using only primary care diagnosis showed similar results as the main analyses (Supplementary Tables 7–10). In a separate sensitivity analysis, we excluded 270 women with prevalent gestational diabetes from the training data and 68 from the test data and obtained similar results to the main findings (Supplementary Tables 11–14). The discrimination and reclassification results were similar when stratifying by sex, however, the predictive performance of each score was slightly higher in females than males (Supplementary Tables 15–19).

## 5 Conclusions

In a UK-based population cohort of ∼202,000 women and men aged 40–69, the original *LRA score,* which consists of simple conventional, non-genetic, risk factors for type 2 diabetes, performed well at predicting 10-year risk of incident type 2 diabetes. There was minimal difference in the discriminative performance of the score when using an expanded definition of ethnicity (the *LRArev score*). However, further incorporation of polygenic risk for type 2 diabetes (the *LRAprs score*) resulted in a moderate improvement in type 2 diabetes 10-year risk prediction compared to the original *LRA score*. The *LRAprs score* also improved the reclassification of individuals into risk groups compared to the *LRA score,* in particular, accurately classifying more cases as ‘high risk’ whilst, as a trade-off, also classifying more non-cases as ‘high risk’.

The *LRA score* was originally derived using a cross-sectional population of 6,390 participants aged 40–75 years recruited from the UK city of Leicester and its surrounding area between 2004 and 2008 and validated in a cross-sectional screening study of 3,171 individuals aged between 40 and 75 years old [12]. Using impaired glucose regulation (also known as prediabetes) or undiagnosed type 2 diabetes as the outcome, the area under the curve (AUC) in the original population was 0.69 (95% CI 0.68, 0.71) and 0.72 (95% CI 0.69, 0.74) in the validation sample. In a separate validation study consisting of 3,203 individuals aged between 50 and 75 years old, the AUC for the *LRA score* and self-reported doctor diagnosis within a 10 year follow-up period was 0.75 (95% CI 0.73, 0.78) [13]. Consequently, the *LRA score* has been recommended by NICE and Diabetes UK as a self-assessment tool to determine future risk of developing type 2 diabetes [15,16]. In the current study, the discriminative performance of the *LRA score* for predicting clinically diagnosed type 2 diabetes within 10 years based on electronic medical records using the test data was found to be higher than previous studies (Harrell's C = 0.796, 95% CI 0.785, 0.806). Furthermore, based on the recommended cut-off of ≥16, both the sensitivity (0.82) and specificity (0.62) of the *LRA score* in the training data was higher in the current study compared to previous findings [12], with the exception of the self-reported type 2 diabetes validation study, which had a higher sensitivity of 0.89, but a much lower specificity of 0.42^13^.

Ethnicity is a strong risk factor for type 2 diabetes, with individuals from a non-White ethnic background having a greater risk and at a younger age compared to those from a White ethnic background [24]. In risk scores, the classification of individuals into an overall ‘non-White’ category does not take into account the heterogeneity in risk within this subgroup. For instance, individuals from a South Asian background have the greatest risk of type 2 diabetes compared to individuals from other ethnic backgrounds [24]. When building the *LRArev* score to expand the categorisation of non-White ethnicity, we also observed that individuals from South Asian ethnic backgrounds had the greatest risk of type 2 diabetes, followed by Other, Black, Mixed and finally, White ethnic groups. Despite this, with a Harrell's C of 0.802 (95% CI 0.792, 0.813) in the test data, the *LRArev score* only minimally improved upon the original *LRA score*. One explanation is that individuals of non-White ethnicity only comprise 4.2% of the analytic sample. Therefore, despite the large variation in type 2 diabetes risk by more refined ethnic subgroup classifications, this does not impact the risk prediction performance of the model when applied to the predominantly White, UKB population.

The incorporation of polygenic risk for the *LRAprs score* resulted in a moderate improvement in risk prediction compared to the *LRA score*, yielding a Harrell's C of 0.829 (95% CI 0.820, 0.839) in the test data. A 2013 systematic review found limited evidence that genetic factors improved existing risk prediction models for type 2 diabetes [25]. However, in recent years an increased number of genetic variants implicated in type 2 diabetes risk have been identified and substantial methodological advances in the development of PRS's have been made [26,27]. Our findings are consistent with more recent studies, which have similarly found that combining both genetic and non-genetic factors results in improved risk prediction of type 2 diabetes [[4], [5], [6], [7], [8], [9], [10], [11]].

We found that incorporating polygenic risk into the original *LRA score* could help identify more individuals at risk of type 2 diabetes at an early stage, albeit with the trade-off that more non-cases will also be classified as high-risk. Whilst promising, these benefits need to be weighed against the increased logistical complexities, economic costs and potential ethical implications of incorporating genetic risk into the existing self-assessed risk score [28]. The *LRA score* was designed to be simple and efficient to complete, requiring individuals to self-complete information that is readily available (i.e. age, sex, ethnicity) or measurable (i.e. waist circumference and BMI). One concern is that the inclusion of genetic risk into a relatively simple score will increase health disparities, in particular in low income settings where polygenic risk scores may be less available, yet the incidence of type 2 diabetes is growing [29]. The disparities could be made worse by adding complexity to a simple score. Nevertheless, a genome array only needs to be performed once in a lifetime, costs have fallen considerably, and PRS for many health conditions can be calculated at minimal additional cost. Investigating the cost-effectiveness of genetic-based risk scores in a diverse variety of settings is an important next step, especially when considering the suitability of using such scores in a clinical setting [30]. A future direction includes using decision curve analysis to investigate how the choice of threshold affects the clinical utility of the risk scores for type 2 diabetes risk prediction.

A strength of the current study is that the UKB cohort collected detailed and standardised genetic and phenotypic data alongside cohort-wide ascertainment of incident type 2 diabetes through ongoing linkage to electronic medical records. The large sample size means that the cohort is well-suited to splitting the dataset into test and training sets, however alternative approaches, such as cross-validation, could be used to internally validate models Type 2 diabetes was determined through multiple sources including primary and secondary care, and death records, and similar findings were observed when restricting analyses to primary care records only.

This study has several limitations. The original *LRA score* was derived using a logistic regression model in cross-sectional data with prediabetes and undiagnosed diabetes, whereas the current study used a different study design and outcome. Nevertheless, the *LRA score* was still highly predictive of type 2 diabetes incidence. Although type 2 diabetes is defined similarly to previous UKB studies [20], this definition has not been externally validated and we cannot rule out some degree of misclassification bias. Previous literature has identified limitations with using NRI as a measure to assess prediction improvement between new and old models, however, bias is reduced when using larger datasets with a small number of predictors [31,32]. Despite reducing the chance of overfitting by splitting the sample into training and test sets, it remains important to externally validate the *LRAprs score* in different populations with varying demographic and lifestyle characteristics. Finally, PRS derived from large datasets specific to non-White ancestries may perform better than the current PRS that was derived from datasets comprised largely of European ancestry participants.

In conclusion, we find that incorporating polygenic risk into the existing *LRA score* moderately improves risk prediction of type 2 diabetes within 10 years. Given that the *LRA score* is widely available as a simple to use risk score, the additional complexities and the cost-benefit trade-offs of including genetic factors into the score requires further exploration.

## Funding

The analyses were funded by the 10.13039/501100000289Cancer Research UK (grant no C16077/A29186), and supported by the 10.13039/501100000769Nuffield Department of Population Health, Oxford University. LJG and KK are supported by the 10.13039/501100000272National Institute for Health and Care Research (NIHR) 10.13039/100000163Applied Research Collaboration East Midlands (ARC EM) and 10.13039/501100000272Leicester NIHR Biomedical Research Centre (BRC). The views expressed are those of the author(s) and not necessarily those of the NIHR or the Department of Health and Social Care. The study funders had no role in the study design; in the collection, analysis, and interpretation of data; in the writing of the report; or in the decision to submit the paper for publication.

## Disclosures

XL, TJL, JB, FB, LC, JAC, and ET have no interests to declare. LJG and KK were members of the research group that developed the original LRA. KK was Chair of the National Institute of Health and Care Excellence Guidelines PH38 Type 2 diabetes: prevention in people at high risk. KK advises Diabetes UK on diabetes related topics including screening for diabetes. KK has acted as a consultant or speaker or received grants for AstraZeneca, Novartis, Novo Nordisk, Sanofi-Aventis, Lilly, Merck Sharp & Dohme, Boehringer Ingelheim, Bayer, Abbott, Amgen, Napp, Roche, Servier, Oramed Pharmaceuticals, and Applied Therapeutics. DJH is Chief Scientific to Our Future Health, a UK Charitable company.

## Data availability

This research has been conducted using the UK Biobank Resource under Application Number 33952. The data reported in this paper are available via application directly to the UK Biobank, https://www.ukbiobank.ac.uk. All code used to set up and run the statistical analyses are available at https://github.com/xiaonanl1996/LRArevprs.

## Author contributions

XL, TJL, LC, JAC, LJG, KK, DJH contributed substantially to the conception and design of the work. DJH acquired the data. XL performed the statistical analysis with code checking performed by JAC. XL and TJL drafted the work. All authors contributed to the interpretation of data, critical revision of the article and provided final approval of the version to be published. All authors agree to be accountable for all aspects of the work in ensuring that questions related to the accuracy or integrity of any part of the work are appropriately investigated and resolved. XL and TJL are the guarantors of this work and, as such, had full access to all the data in the study and take responsibility for the integrity of the data and the accuracy of the data analysis.

## Declaration of competing interest

The authors declare the following financial interests/personal relationships which may be considered as potential competing interests:

LJG and KK were members of the research group that developed the original LRA. KK was Chair of the National Institute of Health and Care Excellence Guidelines PH38 Type 2 diabetes: prevention in people at high risk. KK advises Diabetes UK on diabetes related topics including screening for diabetes. KK has acted as a consultant or speaker or received grants for AstraZeneca, Novartis, Novo Nordisk, Sanofi-Aventis, Lilly, Merck Sharp & Dohme, Boehringer Ingelheim, Bayer, Abbott, Amgen, Napp, Roche, Servier, Oramed Pharmaceuticals, and Applied Therapeutics. DJH is Chief Scientific to Our Future Health, a UK Charitable company.
