# Supplementary material for: Incorporating polygenic risk into the Leicester Risk Assessment score for 10-year risk prediction of type 2 diabetes
Source: Diabetes Metab Syndr. 2024 Apr;18(4):None. doi: 10.1016/j.dsx.2024.102996 (PMC11913737; doi:10.1016/j.dsx.2024.102996)
Supplement: Multimedia component 1 [file mmc1.docx]

**Supplementary Figure 1 -** Flowchart illustrating selection process for final sample size


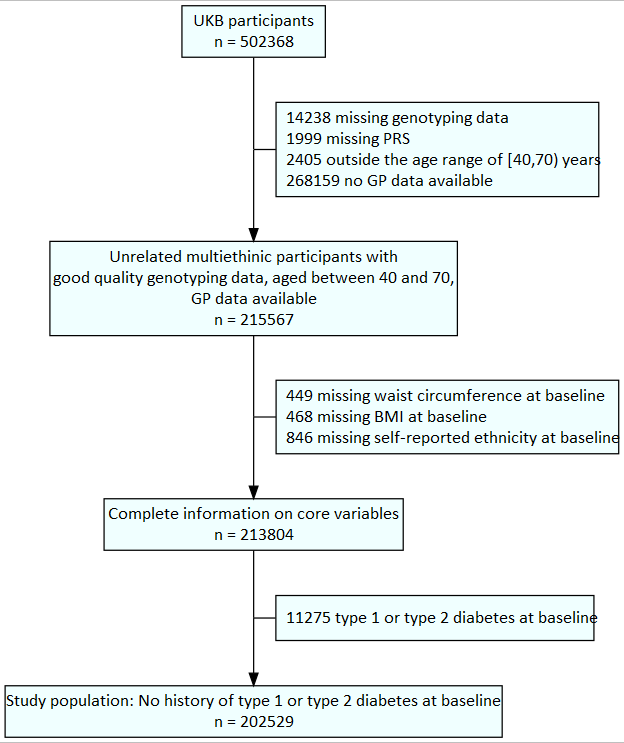


**Supplementary Table 1 -** Clinical codes for defining diabetes and type 2 diabetes.

|  | Source | Clinical codes |
| --- | --- | --- |
| Diabetes diagnosis | Verbal interview diagnosis | 1220, 1223, 1222, 1521 |
|  | ICD-9 | 250* |
|  | ICD-10 | E10*-E14* |
|  | Read version 2 | See column “Prevalent diabetes” in Excel sheet “Dx_Readv2” |
|  | Read version 3 | See column “Prevalent diabetes” in Excel sheet “Dx_Readv3” |
| Diabetes mediations | Verbal interview medication | See column “Diabetes” in Excel sheet “VeI_meds” |
|  | Read version 2 | See column “Diabetes” in Excel sheet “Meds_Readv2” |
|  | BNF | See column “Diabetes” in Excel sheet “Meds_BNF” |
|  | dm+d | See column “Diabetes” in Excel sheet “Meds_dm+d” |
| Type 2 diabetes diagnosis | ICD-10 | E11* |
|  | Read version 2 | See column “T2D” in Excel sheet “Dx_Readv2” |
|  | Read CT version 3 | See column “T2D” in Excel sheet “Dx_Readv3” |

Abbreviations: BNF, British National Formulary, dm+d, Dictionary of Medicines and Devices, ICD, International Classification of Diseases

**Net reclassification index (NRI)**

$$overall NRI={NRI}_{cases}+ {NRI}_{ctrls}$$

$$=\frac{\# cases \uparrow-\# cases\downarrow}{\# cases}+\frac{\# ctrls \downarrow-\#ctrls\uparrow}{\# ctrls}$$

$\uparrow$ represents a change into higher risk group based on the new model and $\downarrow$ means a change in the opposite direction. ${NRI}_{cases}$ represents the proportion of cases that are reclassified to a higher risk group by the new model minus the proportion of cases that are reclassified to a lower risk group. ${NRI}_{ctrls}$ represents the proportion of controls that are reclassified to a lower risk group by the new model minus the proportion of controls that are reclassified to a higher risk group.

**Supplementary Table 2 –** Intercept and model coefficients of the original LRA score.

| **Characteristic** |  | **Coefficients in LRA score** |
| --- | --- | --- |
| Intercept |  | -3.430668 |
| Age in years |  |  |
| 40-49 |  | Reference |
| 50-59 |  | 0.5346095 |
| 60-69 |  | 0.9403614 |
| 70-75 |  | 1.262221 |
| Sex |  |  |
| Male |  | Reference |
| Female |  | 0.0883173 |
| Self-reported ethnicity |  |  |
| White |  | Reference |
| Non-White |  | 0.569434 |
| Family history of diabetes |  |  |
| No |  | Reference |
| Yes |  | 0.465857 |
| Waist circumference in cm |  |  |
| <90 |  | Reference |
| 90-99 |  | 0.4261962 |
| 100-109 |  | 0.5632965 |
| ≥110 |  | 0.8590326 |
| Body Mass Index in kg/m^2^ |  |  |
| <25 |  | Reference |
| 25-29 |  | 0.260582 |
| 30-34 |  | 0.4528574 |
| ≥35 |  | 0.7528332 |
| Self-reported antihypertensive medication use or high blood pressure |  |  |
| No |  | Reference |
| Yes |  | 0.458373 |

**Supplementary Table 3 -** Baseline characteristics of training and test data

| **Characteristics** | **Training, N (%)**  **(N=162,023)** | **Test, N (%) (N=40,506)** | **Total, N (%) (N=202,529)** |
| --- | --- | --- | --- |
| Age in years |  |  |  |
| 40-49 | 38945 (24.0) | 9553 (23.6) | 48498 (23.9) |
| 50-59 | 54971 (33.9) | 13604 (33.6) | 68575 (33.9) |
| 60-69 | 68107 (42.0) | 17349 (42.8) | 85456 (42.2) |
| Female | 89933 (55.5) | 22615 (55.8) | 112548 (55.6) |
| Self-reported ethnicity |  |  |  |
| White | 155205 (95.8) | 38858 (95.9) | 194063 (95.8) |
| Black | 1639 (1.0) | 390 (1.0) | 2029 (1.0) |
| S. Asian | 2353 (1.5) | 572 (1.4) | 2925 (1.4) |
| Mixed | 826 (0.5) | 200 (0.5) | 1026 (0.5) |
| Other | 2000 (1.2) | 486 (1.2) | 2486 (1.2) |
| Family history of diabetes | 32751 (20.2) | 8278 (20.4) | 41029 (20.3) |
| Waist circumference in cm |  |  |  |
| <90 | 82573 (51.0) | 20667 (51.0) | 103240 (51.0) |
| 90-99 | 44419 (27.4) | 11015 (27.2) | 55434 (27.4) |
| 100-109 | 24128 (14.9) | 6113 (15.1) | 30241 (14.9) |
| >=110 | 10903 (6.7) | 2711 (6.7) | 13614 (6.7) |
| Body Mass Index in Kg/m^2^ |  |  |  |
| <25 | 54293 (33.5) | 13586 (33.5) | 67879 (33.5) |
| 25-29 | 69919 (43.2) | 17455 (43.1) | 87374 (43.1) |
| 30-34 | 27852 (17.2) | 6986 (17.2) | 34838 (17.2) |
| >=35 | 9959 (6.1) | 2479 (6.1) | 12438 (6.1) |
| Self-reported antihypertensive medication use or high blood pressure | 43283 (26.7) | 10798 (26.7) | 54081 (26.7) |
| Type 2 diabetes PRS in quintiles |  |  |  |
| 1, lowest | 32405 (20.0) | 8156 (20.1) | 40561 (20.0) |
| 2 | 32404 (20.0) | 8057 (19.9) | 40461 (20.0) |
| 3 | 32405 (20.0) | 8057 (19.9) | 40462 (20.0) |
| 4 | 32404 (20.0) | 8124 (20.1) | 40528 (20.0) |
| 5, highest | 32405 (20.0) | 8112 (20.0) | 40517 (20.0) |

Abbreviations: N, Number, PRS, Polygenic Risk Score

**Supplementary Table 4 -** Sensitivity and specificity computed using the training data

| score | threshold | sensitivity | specificity |
| --- | --- | --- | --- |
| LRA Score | >= 16 | 0.82 | 0.62 |
| LRArev Score | >= 1 | 1.00 | 0.05 |
| LRArev Score | >= 2 | 1.00 | 0.05 |
| LRArev Score | >= 3 | 1.00 | 0.06 |
| LRArev Score | >= 4 | 1.00 | 0.06 |
| LRArev Score | >= 5 | 0.99 | 0.12 |
| LRArev Score | >= 6 | 0.99 | 0.12 |
| LRArev Score | >= 7 | 0.98 | 0.23 |
| LRArev Score | >= 8 | 0.98 | 0.23 |
| LRArev Score | >= 9 | 0.97 | 0.26 |
| LRArev Score | >= 10 | 0.97 | 0.27 |
| LRArev Score | >= 11 | 0.96 | 0.32 |
| LRArev Score | >= 12 | 0.95 | 0.35 |
| LRArev Score | >= 13 | 0.93 | 0.41 |
| LRArev Score | >= 14 | 0.93 | 0.43 |
| LRArev Score | >= 15 | 0.92 | 0.45 |
| LRArev Score | >= 16 | 0.91 | 0.49 |
| LRArev Score | >= 17 | 0.90 | 0.51 |
| LRArev Score | >= 18 | 0.88 | 0.54 |
| LRArev Score | >= 19 | 0.87 | 0.56 |
| LRArev Score | >= 20 | 0.84 | 0.61 |
| LRArev Score | >= 21 | 0.82 | 0.62 |
| LRArev Score | >= 22 | 0.79 | 0.67 |
| LRArev Score | >= 23 | 0.78 | 0.68 |
| LRArev Score | >= 24 | 0.76 | 0.70 |
| LRArev Score | >= 25 | 0.72 | 0.74 |
| LRArev Score | >= 26 | 0.70 | 0.76 |
| LRArev Score | >= 27 | 0.64 | 0.80 |
| LRArev Score | >= 28 | 0.62 | 0.81 |
| LRArev Score | >= 29 | 0.57 | 0.84 |
| LRArev Score | >= 30 | 0.54 | 0.85 |
| LRArev Score | >= 31 | 0.49 | 0.87 |
| LRArev Score | >= 32 | 0.45 | 0.89 |
| LRArev Score | >= 33 | 0.42 | 0.90 |
| LRArev Score | >= 34 | 0.37 | 0.92 |
| LRArev Score | >= 35 | 0.33 | 0.93 |
| LRArev Score | >= 36 | 0.28 | 0.95 |
| LRArev Score | >= 37 | 0.24 | 0.96 |
| LRArev Score | >= 38 | 0.21 | 0.96 |
| LRArev Score | >= 39 | 0.17 | 0.97 |
| LRArev Score | >= 40 | 0.12 | 0.98 |
| LRArev Score | >= 41 | 0.11 | 0.98 |
| LRArev Score | >= 42 | 0.07 | 0.99 |
| LRArev Score | >= 43 | 0.06 | 0.99 |
| LRArev Score | >= 44 | 0.05 | 0.99 |
| LRArev Score | >= 45 | 0.04 | 1.00 |
| LRArev Score | >= 46 | 0.02 | 1.00 |
| LRArev Score | >= 47 | 0.02 | 1.00 |
| LRArev Score | >= 48 | 0.01 | 1.00 |
| LRArev Score | >= 49 | 0.01 | 1.00 |
| LRArev Score | >= 50 | 0.01 | 1.00 |
| LRArev Score | >= 51 | 0.00 | 1.00 |
| LRArev Score | >= 52 | 0.00 | 1.00 |
| LRArev Score | >= 53 | 0.00 | 1.00 |
| LRArev Score | >= 54 | 0.00 | 1.00 |
| LRArev Score | >= 55 | 0.00 | 1.00 |
| LRArev Score | >= 56 | 0.00 | 1.00 |
| LRArev Score | >= 57 | 0.00 | 1.00 |
| LRArev Score | >= 58 | 0.00 | 1.00 |
| LRAprs Score | >= 1 | 1.00 | 0.01 |
| LRAprs Score | >= 2 | 1.00 | 0.01 |
| LRAprs Score | >= 3 | 1.00 | 0.02 |
| LRAprs Score | >= 4 | 1.00 | 0.02 |
| LRAprs Score | >= 5 | 1.00 | 0.03 |
| LRAprs Score | >= 6 | 1.00 | 0.05 |
| LRAprs Score | >= 7 | 1.00 | 0.05 |
| LRAprs Score | >= 8 | 1.00 | 0.07 |
| LRAprs Score | >= 9 | 1.00 | 0.08 |
| LRAprs Score | >= 10 | 0.99 | 0.11 |
| LRAprs Score | >= 11 | 0.99 | 0.13 |
| LRAprs Score | >= 12 | 0.99 | 0.14 |
| LRAprs Score | >= 13 | 0.99 | 0.18 |
| LRAprs Score | >= 14 | 0.99 | 0.20 |
| LRAprs Score | >= 15 | 0.98 | 0.23 |
| LRAprs Score | >= 16 | 0.98 | 0.27 |
| LRAprs Score | >= 17 | 0.98 | 0.28 |
| LRAprs Score | >= 18 | 0.97 | 0.33 |
| LRAprs Score | >= 19 | 0.97 | 0.35 |
| LRAprs Score | >= 20 | 0.96 | 0.38 |
| LRAprs Score | >= 21 | 0.95 | 0.43 |
| LRAprs Score | >= 22 | 0.95 | 0.44 |
| LRAprs Score | >= 23 | 0.93 | 0.48 |
| LRAprs Score | >= 24 | 0.92 | 0.51 |
| LRAprs Score | >= 25 | 0.91 | 0.54 |
| LRAprs Score | >= 26 | 0.89 | 0.58 |
| LRAprs Score | >= 27 | 0.88 | 0.60 |
| LRAprs Score | >= 28 | 0.86 | 0.63 |
| LRAprs Score | >= 29 | 0.84 | 0.66 |
| LRAprs Score | >= 30 | 0.82 | 0.68 |
| LRAprs Score | >= 31 | 0.80 | 0.71 |
| LRAprs Score | >= 32 | 0.78 | 0.73 |
| LRAprs Score | >= 33 | 0.75 | 0.76 |
| LRAprs Score | >= 34 | 0.71 | 0.78 |
| LRAprs Score | >= 35 | 0.69 | 0.80 |
| LRAprs Score | >= 36 | 0.65 | 0.82 |
| LRAprs Score | >= 37 | 0.62 | 0.84 |
| LRAprs Score | >= 38 | 0.59 | 0.86 |
| LRAprs Score | >= 39 | 0.54 | 0.87 |
| LRAprs Score | >= 40 | 0.51 | 0.89 |
| LRAprs Score | >= 41 | 0.46 | 0.90 |
| LRAprs Score | >= 42 | 0.43 | 0.92 |
| LRAprs Score | >= 43 | 0.38 | 0.93 |
| LRAprs Score | >= 44 | 0.35 | 0.94 |
| LRAprs Score | >= 45 | 0.32 | 0.95 |
| LRAprs Score | >= 46 | 0.28 | 0.96 |
| LRAprs Score | >= 47 | 0.25 | 0.96 |
| LRAprs Score | >= 48 | 0.21 | 0.97 |
| LRAprs Score | >= 49 | 0.18 | 0.97 |
| LRAprs Score | >= 50 | 0.15 | 0.98 |
| LRAprs Score | >= 51 | 0.12 | 0.98 |
| LRAprs Score | >= 52 | 0.10 | 0.99 |
| LRAprs Score | >= 53 | 0.09 | 0.99 |
| LRAprs Score | >= 54 | 0.07 | 0.99 |
| LRAprs Score | >= 55 | 0.05 | 0.99 |
| LRAprs Score | >= 56 | 0.04 | 1.00 |
| LRAprs Score | >= 57 | 0.03 | 1.00 |
| LRAprs Score | >= 58 | 0.02 | 1.00 |
| LRAprs Score | >= 59 | 0.02 | 1.00 |
| LRAprs Score | >= 60 | 0.01 | 1.00 |
| LRAprs Score | >= 61 | 0.01 | 1.00 |
| LRAprs Score | >= 62 | 0.01 | 1.00 |
| LRAprs Score | >= 63 | 0.00 | 1.00 |
| LRAprs Score | >= 64 | 0.00 | 1.00 |
| LRAprs Score | >= 65 | 0.00 | 1.00 |
| LRAprs Score | >= 66 | 0.00 | 1.00 |
| LRAprs Score | >= 67 | 0.00 | 1.00 |
| LRAprs Score | >= 68 | 0.00 | 1.00 |
| LRAprs Score | >= 69 | 0.00 | 1.00 |
| LRAprs Score | >= 70 | 0.00 | 1.00 |
| LRAprs Score | >= 71 | 0.00 | 1.00 |
| LRAprs Score | >= 72 | 0.00 | 1.00 |

**Supplementary Figure 2 -** Distribution of scores by risk score and type 2 diabetes status in the training data


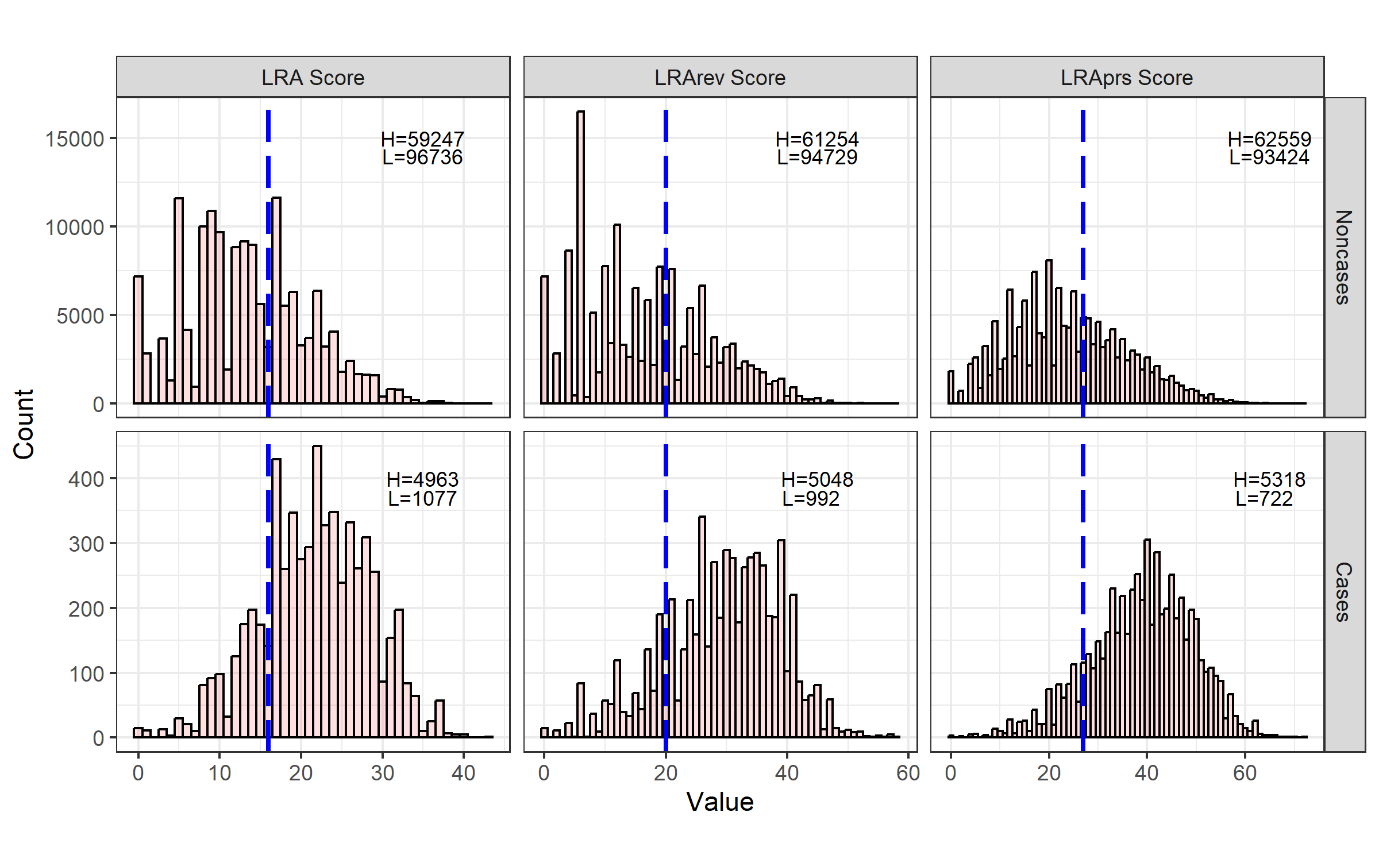


The dashed line represents the optimal cut-off value for each score: 16 for the LRA Score, 20 for the LRArev Score, and 27 for the enhanced score with LRAprs Score. Individuals with risk scores below the cut-off were categorised as “Low risk” and vice versa. Mean scores were 13.8 (SD=7.6), 17.6 (SD=10.9) and 24.6 (SD=12.1) for the *LRA*, *LRArev* and *LRAprs scores*, respectively.

**Supplementary Table 5 -** Harrell’s C statistic for type 2 diabetes using the risk scores in the training data

|  | **Harrell’s C statistic (95% CI)** |
| --- | --- |
| **LRA score** | 0.793 (0.788, 0.798) |
| **LRArev score** | 0.801 (0.796, 0.806) |
| **LRAprs score** | 0.826 (0.821, 0.831) |

Abbreviations: CI, Confidence Interval, PRS, Polygenic Risk Score

**Supplementary Table 6 -** Reclassification tables of A) LRArev score vs LRA score, B) LRAprs score vs LRArev score, and C) the LRAprs score vs LRA score, for risk of type 2 diabetes in the training data

| A) |  | LRArev score | |  |  |
| --- | --- | --- | --- | --- | --- |
|  | **LRA score** | *Low risk* | *High risk* | **NRI (95% CI)** | **Overall NRI (95% CI)** |
| Cases | *Low risk* | 761 | 316 | 0.014  (0.007, 0.022) | 0.001  (-0.006, 0.009) |
|  | *High risk* | 231 | 4732 |  |  |
| Noncases | *Low risk* | 87431 | 9305 | -0.013  (-0.014, -0.011) |  |
|  | *High risk* | 7298 | 51949 |  |  |

| B) |  | LRAprs score | |  |  |
| --- | --- | --- | --- | --- | --- |
|  | **LRArev score** | *Low risk* | *High risk* | **NRI (95% CI)** | **Overall NRI (95% CI)** |
| Cases | *Low risk* | 588 | 404 | 0.045  (0.037, 0.052) | 0.036  (0.029, 0.044) |
|  | *High risk* | 134 | 4914 |  |  |
| Noncases | *Low risk* | 84150 | 10579 | -0.008  (-0.010, -0.007) |  |
|  | *High risk* | 9274 | 51980 |  |  |

| C) |  | LRAprs score | |  |  |
| --- | --- | --- | --- | --- | --- |
|  | **LRA score** | *Low risk* | *High risk* | **NRI (95% CI)** | **Overall NRI (95% CI)** |
| Cases | *Low risk* | 562 | 515 | 0.059  (0.051, 0.067) | 0.038  (0.029, 0.046) |
|  | *High risk* | 160 | 4803 |  |  |
| Noncases | *Low risk* | 83114 | 13622 | -0.021  (-0.023, -0.019) |  |
|  | *High risk* | 10310 | 48937 |  |  |

Abbreviations: NRI, Net Reclassification Index

**Supplementary Figure 3 -** Calibration plot of a) *LRA score,* b) *LRArev score,* and c) *LRAprs score* with 10-year risk of type 2 diabetes using test data. Since the maximum follow-up for one quintile group was less than 10 years, we opted to compute the observed 9-year risk as an approximation of the observed 10-year risk (i.e. y-axis “Observed risk” in all subplots below shows the observed 9-year risk and x-axis “Predicted risk” shows the Leicester score or predicted 10-year risk).

a) b)


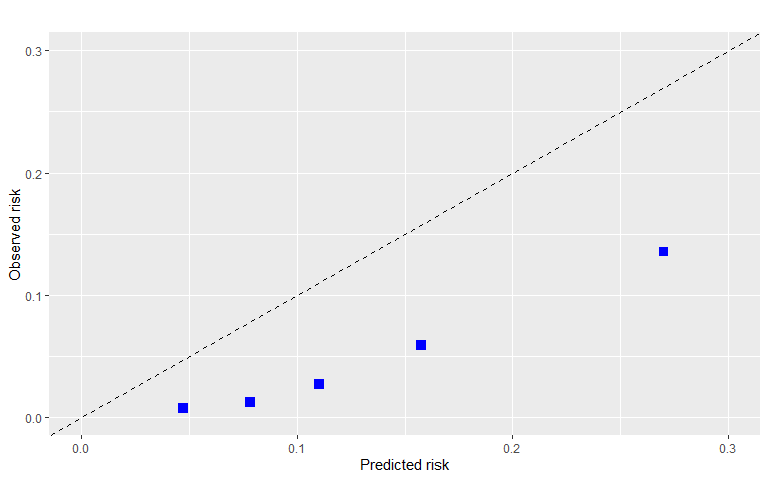

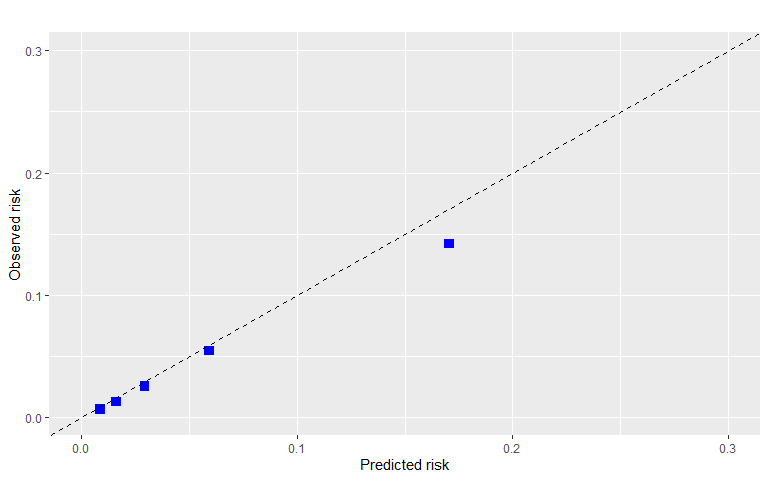


c)


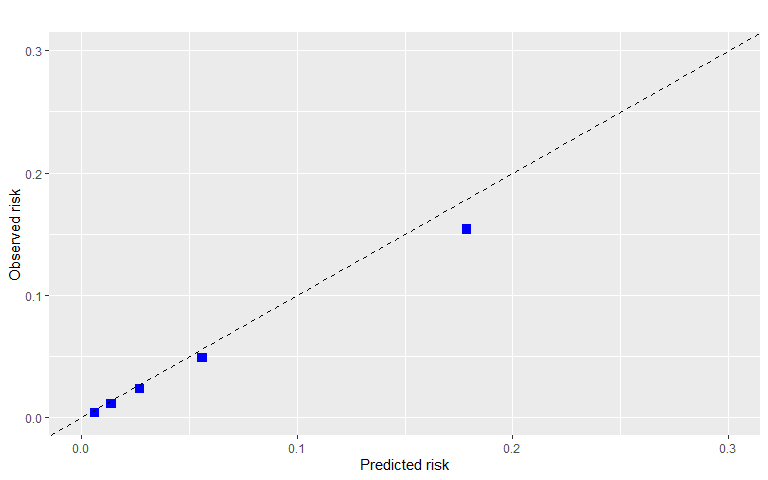


**Supplementary Figure 4 –** Venn diagram of incident type 2 diabetes identified across different sources in the training data. HES, hospital inpatient; Dth, death registry; GP, primary care data.


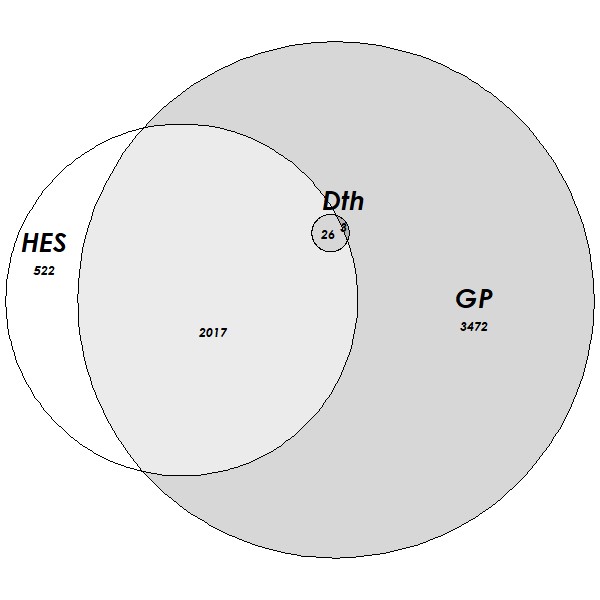


**Supplementary Table 7 -** Cox proportional-hazards models for type 2 diabetes derived using primary care diagnosis only in the training dataset and scoring system for risk scores

| **Characteristic** |  | **LRA score** |  | **LRArev score** | | |  | **LRAprs score** | | |
| --- | --- | --- | --- | --- | --- | --- | --- | --- | --- | --- |
|  |  | **Score^a^** |  | **HR (95% CI)** | **β (95% CI)^b^** | **Score** |  | **HR (95% CI)** | **β (95% CI) ^b^** | **Score** |
| Age in years**^c^** |  |  |  |  |  |  |  |  |  |  |
| 40-49 |  | 0 |  | Reference | Reference | 0 |  | Reference | Reference | 0 |
| 50-59 |  | 5 |  | 1.44 (1.33, 1.57) | 0.37 (0.28, 0.45) | 4 |  | 1.50 (1.38, 1.63) | 0.41 (0.33, 0.49) | 4 |
| 60-69 |  | 9 |  | 1.80 (1.66, 1.95) | 0.59 (0.51, 0.67) | 6 |  | 1.94 (1.79, 2.11) | 0.66 (0.58, 0.74) | 7 |
| Sex |  |  |  |  |  |  |  |  |  |  |
| Female |  | 0 |  | Reference | Reference | 0 |  | Reference | Reference | 0 |
| Male |  | 1 |  | 1.19 (1.12, 1.27) | 0.17 (0.11, 0.24) | 2 |  | 1.22 (1.14, 1.30) | 0.20 (0.13, 0.26) | 2 |
| Self-reported ethnicity |  |  |  |  |  |  |  |  |  |  |
| White |  | 0 |  | Reference | Reference | 0 |  | Reference | Reference | 0 |
| Non-White |  | 6 |  | - | - | - |  | - | - | - |
| Black |  | - |  | 2.36 (1.96, 2.83) | 0.86 (0.68, 1.04) | 9 |  | 2.38 (1.98, 2.85) | 0.87 (0.68, 1.05) | 9 |
| South Asian |  | - |  | 3.84 (3.39, 4.36) | 1.35 (1.22, 1.47) | 13 |  | 4.06 (3.58, 4.61) | 1.40 (1.27, 1.53) | 14 |
| Mixed |  | - |  | 1.92 (1.40, 2.63) | 0.65 (0.34, 0.97) | 7 |  | 1.85 (1.35, 2.53) | 0.61 (0.30, 0.93) | 6 |
| Other |  | - |  | 2.85 (2.40, 3.38) | 1.05 (0.88, 1.22) | 10 |  | 2.92 (2.46, 3.47) | 1.07 (0.90, 1.24) | 11 |
| Family history of diabetes |  |  |  |  |  |  |  |  |  |  |
| No |  | 0 |  | Reference | Reference | 0 |  | Reference | Reference | 0 |
| Yes |  | 5 |  | 1.89 (1.78, 2.00) | 0.64 (0.58, 0.69) | 6 |  | 1.67 (1.58, 1.77) | 0.51 (0.45, 0.57) | 5 |
| Waist circumference in cm |  |  |  |  |  |  |  |  |  |  |
| <90 |  | 0 |  | Reference | Reference | 0 |  | Reference | Reference | 0 |
| 90-99 |  | 4 |  | 1.96 (1.77, 2.17) | 0.67 (0.57, 0.77) | 7 |  | 1.91 (1.73, 2.11) | 0.65 (0.55, 0.75) | 6 |
| 100-109 |  | 6 |  | 3.17 (2.83, 3.56) | 1.15 (1.04, 1.27) | 12 |  | 3.07 (2.74, 3.44) | 1.12 (1.01, 1.24) | 11 |
| ≥110 |  | 9 |  | 4.52 (3.94, 5.19) | 1.51 (1.37, 1.65) | 15 |  | 4.40 (3.84, 5.05) | 1.48 (1.35, 1.62) | 15 |
| Body Mass Index |  |  |  |  |  |  |  |  |  |  |
| <25 |  | 0 |  | Reference | Reference | 0 |  | Reference | Reference | 0 |
| 25-29 |  | 3 |  | 2.02 (1.79, 2.27) | 0.70 (0.58, 0.82) | 7 |  | 1.92 (1.70, 2.17) | 0.65 (0.53, 0.77) | 7 |
| 30-34 |  | 5 |  | 3.20 (2.79, 3.66) | 1.16 (1.03, 1.30) | 12 |  | 2.97 (2.59, 3.40) | 1.09 (0.95, 1.22) | 11 |
| ≥35 |  | 8 |  | 4.36 (3.73, 5.10) | 1.47 (1.32, 1.63) | 15 |  | 4.04 (3.45, 4.73) | 1.40 (1.24, 1.55) | 14 |
| Self-reported antihypertensive medication use or high blood pressure |  |  |  |  |  |  |  |  |  |  |
| No |  | 0 |  | Reference | Reference | 0 |  | Reference | Reference | 0 |
| Yes |  | 5 |  | 1.70 (1.61, 1.80) | 0.53 (0.48, 0.59) | 5 |  | 1.64 (1.56, 1.74) | 0.50 (0.44, 0.55) | 5 |
| Type 2 diabetes PRS in quintiles |  |  |  |  |  |  |  |  |  |  |
| 1, lowest |  | - |  |  |  | - |  | Reference | Reference | 0 |
| 2 |  | - |  |  |  | - |  | 1.68 (1.48, 1.92) | 0.52 (0.39, 0.65) | 5 |
| 3 |  | - |  |  |  | - |  | 2.38 (2.10, 2.69) | 0.87 (0.74, 0.99) | 9 |
| 4 |  | - |  |  |  | - |  | 3.21 (2.85, 3.62) | 1.17 (1.05, 1.29) | 12 |
| 5, highest |  | - |  |  |  | - |  | 5.13 (4.58, 5.76) | 1.64 (1.52, 1.75) | 16 |

Abbreviations: CI, Confidence Interval, PRS, Polygenic Risk Score, HR, Hazard Ratio

**^a^** The scoring system of used for the *LRA score* was originally published elsewhere^4^

**^b^** The β is the log(Hazard ratio) and all categories within each variable were statistically significantly associated with type 2 diabetes risk (p<0.001).

**^c^** The original Leicester score includes a score of 13 assigned to 70-75 year olds. As the upper age limit in UKB is 69 years, this age group was not included in the model.

**Supplementary Table 8 -** Harrell’s C statistic for type 2 diabetes derived using primary care diagnoses only using the risk scores in the training and test data

|  | **Harrell’s C statistic (95% CI)** | |
| --- | --- | --- |
|  | **Training data** | **Test data** |
| **LRA score** | 0.797 (0.792, 0.803) | 0.800 (0.789, 0.811) |
| **LRArev score** | 0.807 (0.802, 0.812) | 0.809 (0.798, 0.819) |
| **LRAprs score** | 0.833 (0.828, 0.838) | 0.839 (0.829, 0.848) |

Abbreviations: CI, Confidence Interval, PRS, Polygenic Risk Score

**Supplementary Table 9 -** Reclassification tables of **A)** *LRArev score* vs *LRA score*, **B)** *LRAprs score* vs *LRArev score*, and **C)** the *LRAprs score* vs *LRA score*, for risk of type 2 diabetes derived using primary care diagnosis only in the training data. We used 16, 21, and 29 as the cut-offs for *LRA, LRArev*, and *LRAprs scores* respectively.

| A) |  | LRArev score | |  |  |
| --- | --- | --- | --- | --- | --- |
|  | **LRA score** | *Low risk* | *High risk* | **NRI (95% CI)** | **Overall NRI (95% CI)** |
| Cases | *Low risk* | 638 | 309 | 0.016  (0.007, 0.024) | 0.004  (-0.004, 0.012) |
|  | *High risk* | 222 | 4349 |  |  |
| Noncases | *Low risk* | 87047 | 9819 | -0.012  (-0.013, -0.010) |  |
|  | *High risk* | 7998 | 51641 |  |  |

| B) |  | LRAprs score | |  |  |
| --- | --- | --- | --- | --- | --- |
|  | **LRArev score** | *Low risk* | *High risk* | **NRI (95% CI)** | **Overall NRI (95% CI)** |
| Cases | *Low risk* | 494 | 366 | 0.047  (0.038, 0.054) | 0.032  (0.024, 0.040) |
|  | *High risk* | 109 | 4549 |  |  |
| Noncases | *Low risk* | 83997 | 11048 | -0.014  (-0.016, -0.012) |  |
|  | *High risk* | 8825 | 52635 |  |  |

| C) |  | LRAprs score | |  |  |
| --- | --- | --- | --- | --- | --- |
|  | **LRA score** | *Low risk* | *High risk* | **NRI (95% CI)** | **Overall NRI (95% CI)** |
| Cases | *Low risk* | 469 | 478 | 0.062  (0.054, 0.071) | 0.037  (0.028, 0.045) |
|  | *High risk* | 134 | 4437 |  |  |
| Noncases | *Low risk* | 82796 | 14070 | -0.026  (-0.028, -0.024) |  |
|  | *High risk* | 10026 | 49613 |  |  |

Abbreviations: NRI, Net Reclassification Index

**Supplementary Table 10 -** Reclassification tables of **A)** *LRArev score* vs *LRA score*, **B)** *LRAprs score* vs *LRArev score*, and **C)** the *LRAprs score* vs *LRArev score*, for risk of type 2 diabetes derived using primary care diagnosis only in the test data. We used 16, 21, and 29 as the cut-offs for *LRA, LRArev*, and *LRAprs scores* respectively.

| A) |  | LRArev score | |  |  |
| --- | --- | --- | --- | --- | --- |
|  | **LRA score** | *Low risk* | *High risk* | **NRI (95% CI)** | **Overall NRI (95% CI)** |
| Cases | *Low risk* | 152 | 66 | 0.012  (-0.005, 0.029) | -0.002  (-0.020, 0.015) |
|  | *High risk* | 50 | 1036 |  |  |
| Noncases | *Low risk* | 21750 | 2473 | -0.014  (-0.018, -0.011) |  |
|  | *High risk* | 1906 | 13073 |  |  |

| B) |  | LRAprs score | |  |  |
| --- | --- | --- | --- | --- | --- |
|  | **LRArev score** | *Low risk* | *High risk* | **NRI (95% CI)** | **Overall NRI (95% CI)** |
| Cases | *Low risk* | 113 | 89 | 0.054  (0.038, 0.069) | 0.041  (0.025, 0.056) |
|  | *High risk* | 19 | 1083 |  |  |
| Noncases | *Low risk* | 20962 | 2694 | -0.013  (-0.016, -0.009) |  |
|  | *High risk* | 2199 | 13347 |  |  |

| C) |  | LRAprs score | |  |  |
| --- | --- | --- | --- | --- | --- |
|  | **LRA score** | *Low risk* | *High risk* | **NRI (95% CI)** | **Overall NRI (95% CI)** |
| Cases | *Low risk* | 110 | 108 | 0.066  (0.050, 0.082) | 0.039  (0.021, 0.056) |
|  | *High risk* | 22 | 1064 |  |  |
| Noncases | *Low risk* | 20657 | 3566 | -0.027  (-0.031, -0.023) |  |
|  | *High risk* | 2504 | 12475 |  |  |

Abbreviations: NRI, Net Reclassification Index

**Supplementary Table 11-** Cox proportional-hazards models for type 2 diabetes in the training dataset and scoring system for risk scores after further excluding prevalent gestational diabetes (N=161753)

| **Characteristic** |  | **LRA score** |  | **LRArev score** | | |  | **LRAprs score** | | |
| --- | --- | --- | --- | --- | --- | --- | --- | --- | --- | --- |
|  |  | **Score** |  | **HR (95% CI)** | **β (95% CI)** | **Score** |  | **HR (95% CI)** | **β (95% CI)** | **Score** |
| Age in years |  |  |  |  |  |  |  |  |  |  |
| 40-49 |  | 0 |  | Reference | Reference | 0 |  | Reference | Reference | 0 |
| 50-59 |  | 5 |  | 1.49 (1.37, 1.61) | 0.40 (0.32, 0.48) | 4 |  | 1.55 (1.43, 1.67) | 0.44 (0.35, 0.52) | 4 |
| 60-69 |  | 9 |  | 1.91 (1.77, 2.07) | 0.65 (0.57, 0.73) | 6 |  | 2.05 (1.90, 2.22) | 0.72 (0.64, 0.80) | 7 |
| Sex |  |  |  |  |  |  |  |  |  |  |
| Female |  | 0 |  | Reference | Reference | 0 |  | Reference | Reference | 0 |
| Male |  | 1 |  | 1.18 (1.10, 1.25) | 0.16 (0.10, 0.22) | 2 |  | 1.20 (1.13, 1.28) | 0.18 (0.12, 0.25) | 2 |
| Self-reported ethnicity |  |  |  |  |  |  |  |  |  |  |
| White |  | 0 |  | Reference | Reference | 0 |  | Reference | Reference | 0 |
| Non-White |  | 6 |  | - | - | - |  | - | - | - |
| Black |  | - |  | 2.31 (1.93, 2.76) | 0.84 (0.66, 1.02) | 8 |  | 2.34 (1.96, 2.80) | 0.85 (0.67, 1.03) | 9 |
| South Asian |  | - |  | 3.83 (3.39, 4.33) | 1.34 (1.22, 1.47) | 13 |  | 4.04 (3.58, 4.57) | 1.40 (1.28, 1.52) | 14 |
| Mixed |  | - |  | 2.09 (1.55, 2.80) | 0.73 (0.44, 1.03) | 7 |  | 2.02 (1.51, 2.71) | 0.70 (0.41, 1.00) | 7 |
| Other |  | - |  | 2.79 (2.37, 3.30) | 1.03 (0.86, 1.19) | 10 |  | 2.86 (2.42, 3.38) | 1.05 (0.89, 1.22) | 11 |
| Family history of diabetes |  |  |  |  |  |  |  |  |  |  |
| No |  | 0 |  | Reference | Reference | 0 |  | Reference | Reference | 0 |
| Yes |  | 5 |  | 1.89 (1.79, 1.99) | 0.63 (0.58, 0.69) | 6 |  | 1.68 (1.59, 1.77) | 0.52 (0.46, 0.57) | 5 |
| Waist circumference in cm |  |  |  |  |  |  |  |  |  |  |
| <90 |  | 0 |  | Reference | Reference | 0 |  | Reference | Reference | 0 |
| 90-99 |  | 4 |  | 1.93 (1.75, 2.12) | 0.66 (0.56, 0.75) | 7 |  | 1.88 (1.71, 2.07) | 0.63 (0.54, 0.73) | 6 |
| 100-109 |  | 6 |  | 3.15 (2.82, 3.51) | 1.15 (1.04, 1.26) | 11 |  | 3.05 (2.74, 3.40) | 1.12 (1.01, 1.22) | 11 |
| ≥110 |  | 9 |  | 4.48 (3.93, 5.11) | 1.50 (1.37, 1.63) | 15 |  | 4.37 (3.83, 4.98) | 1.47 (1.34, 1.60) | 15 |
| Body Mass Index in kg/m^2^ |  |  |  |  |  |  |  |  |  |  |
| <25 |  | 0 |  | Reference | Reference | 0 |  | Reference | Reference | 0 |
| 25-29 |  | 3 |  | 1.83 (1.63, 2.04) | 0.60 (0.49, 0.71) | 6 |  | 1.74 (1.56, 1.95) | 0.56 (0.45, 0.67) | 6 |
| 30-34 |  | 5 |  | 2.80 (2.47, 3.18) | 1.03 (0.90, 1.16) | 10 |  | 2.61 (2.30, 2.96) | 0.96 (0.83, 1.09) | 10 |
| ≥35 |  | 8 |  | 3.86 (3.33, 4.48) | 1.35 (1.20, 1.50) | 14 |  | 3.59 (3.10, 4.16) | 1.28 (1.13, 1.43) | 13 |
| Self-reported antihypertensive medication use or high blood pressure |  |  |  |  |  |  |  |  |  |  |
| No |  | 0 |  | Reference | Reference | 0 |  | Reference | Reference | 0 |
| Yes |  | 5 |  | 1.70 (1.61, 1.79) | 0.53 (0.47, 0.58) | 5 |  | 1.64 (1.56, 1.73) | 0.50 (0.44, 0.55) | 5 |
| Type 2 diabetes PRS in quintiles |  |  |  |  |  |  |  |  |  |  |
| 1, lowest |  | - |  |  |  | - |  | Reference | Reference | 0 |
| 2 |  | - |  |  |  | - |  | 1.63 (1.44, 1.84) | 0.49 (0.37, 0.61) | 5 |
| 3 |  | - |  |  |  | - |  | 2.22 (1.98, 2.49) | 0.80 (0.68, 0.91) | 8 |
| 4 |  | - |  |  |  | - |  | 2.93 (2.63, 3.28) | 1.08 (0.97, 1.19) | 11 |
| 5, highest |  | - |  |  |  | - |  | 4.65 (4.18, 5.16) | 1.54 (1.43, 1.64) | 15 |

**Supplementary Table 12 -** Harrell’s C statistic for type 2 diabetes using the risk scores in the training and test data after excluding prevalent gestational diabetes diagnoses

|  | **Harrell’s C statistic (95% CI)** | |
| --- | --- | --- |
|  | **Training data** | **Test data** |
| **LRA score** | 0.794 (0.789, 0.799) | 0.798 (0.787, 0.808) |
| **LRArev score** | 0.802 (0.797, 0.807) | 0.804 (0.794, 0.815) |
| **LRAprs score** | 0.826 (0.821, 0.831) | 0.830 (0.821, 0.840) |

Abbreviations: CI, Confidence Interval, PRS, Polygenic Risk Score

**Supplementary Table 13 -** Reclassification tables of **A)** *LRArev score* vs *LRA score*, **B)** *LRAprs score* vs *LRArev score*, and **C)** the *LRAprs score* vs *LRA score*, for risk of type 2 diabetes in the training data after further excluding prevalent gestational diabetes. We used 16, 20, and 28 as the cut-offs for *LRA, LRArev*, and *LRAprs scores* respectively.

| A) |  | LRArev score | |  |  |
| --- | --- | --- | --- | --- | --- |
|  | **LRA score** | *Low risk* | *High risk* | **NRI (95% CI)** | **Overall NRI (95% CI)** |
| Cases | *Low risk* | 764 | 302 | 0.011  (0.004, 0.019) | 0.005  (-0.003, 0.013) |
|  | *High risk* | 234 | 4710 |  |  |
| Noncases | *Low risk* | 88187 | 8386 | -0.007  (-0.008, -0.005) |  |
|  | *High risk* | 7329 | 51841 |  |  |

| B) |  | LRAprs score | |  |  |
| --- | --- | --- | --- | --- | --- |
|  | **LRArev score** | *Low risk* | *High risk* | **NRI (95% CI)** | **Overall NRI (95% CI)** |
| Cases | *Low risk* | 621 | 377 | 0.042  (0.034, 0.048) | 0.034  (0.026, 0.041) |
|  | *High risk* | 127 | 4885 |  |  |
| Noncases | *Low risk* | 85560 | 9956 | -0.008  (-0.010, -0.006) |  |
|  | *High risk* | 8727 | 51500 |  |  |

| C) |  | LRAprs score | |  |  |
| --- | --- | --- | --- | --- | --- |
|  | **LRA score** | *Low risk* | *High risk* | **NRI (95% CI)** | **Overall NRI (95% CI)** |
| Cases | *Low risk* | 587 | 479 | 0.053  (0.044, 0.061) | 0.038  (0.030, 0.047) |
|  | *High risk* | 161 | 4783 |  |  |
| Noncases | *Low risk* | 83907 | 12666 | -0.015  (-0.016, -0.013) |  |
|  | *High risk* | 10380 | 48790 |  |  |

Abbreviations: NRI, Net Reclassification Index

**Supplementary Table 14 -** Reclassification tables of **A)** *LRArev score* vs *LRA score*, **B)** *LRAprs score* vs *LRArev score*, and **C)** the *LRAprs score* vs *LRA score*, for risk of type 2 diabetes in the test data after further excluding prevalent gestational diabetes. We used 16, 20, and 28 as the cut-offs for *LRA, LRArev*, and *LRAprs scores* respectively.

| A) |  | LRArev score | |  |  |
| --- | --- | --- | --- | --- | --- |
|  | **LRA score** | *Low risk* | *High risk* | **NRI (95% CI)** | **Overall NRI (95% CI)** |
| Cases | *Low risk* | 188 | 60 | 0.004  (-0.012, 0.018) | -0.006  (-0.022, 0.009) |
|  | *High risk* | 55 | 1122 |  |  |
| Noncases | *Low risk* | 22032 | 2114 | -0.010  (-0.013, -0.006) |  |
|  | *High risk* | 1740 | 13127 |  |  |

| B) |  | LRAprs score | |  |  |
| --- | --- | --- | --- | --- | --- |
|  | **LRArev score** | *Low risk* | *High risk* | **NRI (95% CI)** | **Overall NRI (95% CI)** |
| Cases | *Low risk* | 151 | 92 | 0.046  (0.032, 0.061) | 0.040  (0.025, 0.055) |
|  | *High risk* | 26 | 1156 |  |  |
| Noncases | *Low risk* | 21337 | 2435 | -0.007  (-0.010, -0.003) |  |
|  | *High risk* | 2177 | 13064 |  |  |

| C) |  | LRAprs score | |  |  |
| --- | --- | --- | --- | --- | --- |
|  | **LRA score** | *Low risk* | *High risk* | **NRI (95% CI)** | **Overall NRI (95% CI)** |
| Cases | *Low risk* | 141 | 107 | 0.050  (0.034, 0.066) | 0.034  (0.016, 0.051) |
|  | *High risk* | 36 | 1141 |  |  |
| Noncases | *Low risk* | 20937 | 3209 | -0.016  (-0.020, -0.012) |  |
|  | *High risk* | 2577 | 12290 |  |  |

Abbreviations: NRI, Net Reclassification Index

**Supplementary Table 15 -** Harrell’s C statistic for type 2 diabetes using the risk scores in the training and test data for females and males separately

|  | **Harrell’s C statistic (95% CI)** | |
| --- | --- | --- |
|  | **Training data** | **Test data** |
| **Females** |  |  |
| *LRA score* | 0.810 (0.802, 0.818) | 0. 825 (0.809, 0.840) |
| *LRArev score* | 0.816 (0.808, 0.824) | 0.828 (0.813, 0.843) |
| *LRAprs score* | 0.837 (0.829, 0.844) | 0.850 (0.835, 0.864) |
| **Males** |  |  |
| *LRA score* | 0.761 (0.753, 0.768) | 0.752 (0.736, 0.767) |
| *LRArev score* | 0.774 (0.767, 0.781) | 0.762 (0.747, 0.778) |
| *LRAprs score* | 0.803 (0.796, 0.809) | 0.797 (0.783, 0.811) |

Abbreviations: CI, Confidence Interval, PRS, Polygenic Risk Score

**Supplementary Table 16 -** Reclassification tables of **A)** *LRArev score* vs *LRA score*, **B)** *LRAprs score* vs *LRArev score*, and **C)** the *LRAprs score* vs *LRA score*, for risk of type 2 diabetes in the training data for females. We used 16, 20, and 28 as the cut-offs for *LRA, LRArev*, and *LRAprs scores* respectively.

| A) |  | LRArev score | |  |  |
| --- | --- | --- | --- | --- | --- |
|  | **LRA score** | *Low risk* | *High risk* | **NRI (95% CI)** | **Overall NRI (95% CI)** |
| Cases | *Low risk* | 466 | 111 | -0.035  (-0.049,-0.022) | 0.002  (-0.012,0.017) |
|  | *High risk* | 200 | 1734 |  |  |
| Noncases | *Low risk* | 58982 | 3155 | 0.038  (0.035,0.040) |  |
|  | *High risk* | 6454 | 18831 |  |  |

| B) |  | LRAprs score | |  |  |
| --- | --- | --- | --- | --- | --- |
|  | **LRArev score** | *Low risk* | *High risk* | **NRI (95% CI)** | **Overall NRI (95% CI)** |
| Cases | *Low risk* | 398 | 268 | 0.090 (0.078, 0.103) | 0.046 (0.033, 0.060) |
|  | *High risk* | 43 | 1802 |  |  |
| Noncases | *Low risk* | 58918 | 6518 | -0.044 (-0.046, -0.041) |  |
|  | *High risk* | 2697 | 19289 |  |  |

| C) |  | LRAprs score | |  |  |
| --- | --- | --- | --- | --- | --- |
|  | **LRA score** | *Low risk* | *High risk* | **NRI (95% CI)** | **Overall NRI (95% CI)** |
| Cases | *Low risk* | 348 | 229 | 0.054 (0.040, 0.068) | 0.048 (0.034, 0.062) |
|  | *High risk* | 93 | 1841 |  |  |
| Noncases | *Low risk* | 56164 | 5973 | -0.006 (-0.008, -0.004) |  |
|  | *High risk* | 5451 | 19834 |  |  |

Abbreviations: NRI, Net Reclassification Index

**Supplementary Table 17 -** Reclassification tables of **A)** *LRArev score* vs *LRA score*, **B)** *LRAprs score* vs *LRArev score*, and **C)** the *LRAprs score* vs *LRA score*, for risk of type 2 diabetes in the training data for males. We used 16, 20, and 28 as the cut-offs for *LRA, LRArev*, and *LRAprs scores* respectively.

| A) |  | LRArev score | |  |  |
| --- | --- | --- | --- | --- | --- |
|  | **LRA score** | *Low risk* | *High risk* | **NRI (95% CI)** | **Overall NRI (95% CI)** |
| Cases | *Low risk* | 295 | 205 | 0.049 (0.041, 0.058) | -0.028 (-0.036, -0.019) |
|  | *High risk* | 31 | 2998 |  |  |
| Noncases | *Low risk* | 28449 | 6150 | -0.077 (-0.080, -0.075) |  |
|  | *High risk* | 844 | 33118 |  |  |

| B) |  | LRAprs score | |  |  |
| --- | --- | --- | --- | --- | --- |
|  | **LRArev score** | *Low risk* | *High risk* | **NRI (95% CI)** | **Overall NRI (95% CI)** |
| Cases | *Low risk* | 190 | 136 | 0.013 (0.005, 0.021) | 0.049 (0.041, 0.059) |
|  | *High risk* | 91 | 3112 |  |  |
| Noncases | *Low risk* | 25232 | 4061 | 0.037 (0.034, 0.040) |  |
|  | *High risk* | 6577 | 32691 |  |  |

| C) |  | LRAprs score | |  |  |
| --- | --- | --- | --- | --- | --- |
|  | **LRA score** | *Low risk* | *High risk* | **NRI (95% CI)** | **Overall NRI (95% CI)** |
| Cases | *Low risk* | 214 | 286 | 0.062 (0.052, 0.072) | 0.021 (0.011, 0.031) |
|  | *High risk* | 67 | 2962 |  |  |
| Noncases | *Low risk* | 26950 | 7649 | -0.041 (-0.044, -0.038) |  |
|  | *High risk* | 4859 | 29103 |  |  |

Abbreviations: NRI, Net Reclassification Index

**Supplementary Table 18 -** Reclassification tables of **A)** *LRArev score* vs *LRA score*, **B)** *LRAprs score* vs *LRArev score*, and **C)** the *LRAprs score* vs *LRA score*, for risk of type 2 diabetes in the test data for females. We used 16, 20, and 28 as the cut-offs for *LRA, LRArev*, and *LRAprs scores* respectively.

| A) |  | LRArev score | |  |  |
| --- | --- | --- | --- | --- | --- |
|  | **LRA score** | *Low risk* | *High risk* | **NRI (95% CI)** | **Overall NRI (95% CI)** |
| Cases | *Low risk* | 107 | 22 | -0.045 (-0.073, -0.018) | -0.010 (-0.038, 0.017) |
|  | *High risk* | 49 | 424 |  |  |
| Noncases | *Low risk* | 14938 | 786 | 0.035 (0.030, 0.039) |  |
|  | *High risk* | 1551 | 4738 |  |  |

| B) |  | LRAprs score | |  |  |
| --- | --- | --- | --- | --- | --- |
|  | **LRArev score** | *Low risk* | *High risk* | **NRI (95% CI)** | **Overall NRI (95% CI)** |
| Cases | *Low risk* | 97 | 59 | 0.083 (0.058, 0.110) | 0.039 (0.014, 0.067) |
|  | *High risk* | 9 | 437 |  |  |
| Noncases | *Low risk* | 14836 | 1653 | -0.044 (-0.048, -0.040) |  |
|  | *High risk* | 683 | 4841 |  |  |

| C) |  | LRAprs score | |  |  |
| --- | --- | --- | --- | --- | --- |
|  | **LRA score** | *Low risk* | *High risk* | **NRI (95% CI)** | **Overall NRI (95% CI)** |
| Cases | *Low risk* | 84 | 45 | 0.038 (0.012, 0.066) | 0.029 (0.003, 0.056) |
|  | *High risk* | 22 | 451 |  |  |
| Noncases | *Low risk* | 14177 | 1547 | -0.009 (-0.014, -0.005) |  |
|  | *High risk* | 1342 | 4947 |  |  |

Abbreviations: NRI, Net Reclassification Index

**Supplementary Table 19 -** Reclassification tables of **A)** *LRArev score* vs *LRA score*, **B)** *LRAprs score* vs *LRArev score*, and **C)** the *LRAprs score* vs *LRA score*, for risk of type 2 diabetes in the test data for males. We used 16, 20, and 28 as the cut-offs for *LRA, LRArev*, and *LRAprs scores* respectively.

| A) |  | LRArev score | |  |  |
| --- | --- | --- | --- | --- | --- |
|  | **LRA score** | *Low risk* | *High risk* | **NRI (95% CI)** | **Overall NRI (95% CI)** |
| Cases | *Low risk* | 79 | 45 | 0.048 (0.032, 0.064) | -0.032 (-0.049, -0.015) |
|  | *High risk* | 5 | 705 |  |  |
| Noncases | *Low risk* | 6920 | 1544 | -0.080 (-0.084, -0.075) |  |
|  | *High risk* | 182 | 8411 |  |  |

| B) |  | LRAprs score | |  |  |
| --- | --- | --- | --- | --- | --- |
|  | **LRArev score** | *Low risk* | *High risk* | **NRI (95% CI)** | **Overall NRI (95% CI)** |
| Cases | *Low risk* | 48 | 36 | 0.023 (0.006, 0.042) | 0.060 (0.042, 0.081) |
|  | *High risk* | 17 | 733 |  |  |
| Noncases | *Low risk* | 6123 | 979 | 0.037 (0.031, 0.043) |  |
|  | *High risk* | 1618 | 8337 |  |  |

| C) |  | LRAprs score | |  |  |
| --- | --- | --- | --- | --- | --- |
|  | **LRA score** | *Low risk* | *High risk* | **NRI (95% CI)** | **Overall NRI (95% CI)** |
| Cases | *Low risk* | 52 | 72 | 0.071 (0.050, 0.091) | 0.028 (0.007, 0.051) |
|  | *High risk* | 13 | 697 |  |  |
| Noncases | *Low risk* | 6526 | 1938 | -0.042 (-0.049, -0.036) |  |
|  | *High risk* | 1215 | 7378 |  |  |

Abbreviations: NRI, Net Reclassification Index
